# Tailoring Natural and Fly Ash-Based Zeolites Surfaces for Efficient 2,4-D Herbicide Adsorption: The Role of Hexadecyltrimethylammonium Bromide Modification

**DOI:** 10.3390/molecules29225244

**Published:** 2024-11-06

**Authors:** Agata Jankowska, Rafał Panek, Wojciech Franus, Joanna Goscianska

**Affiliations:** 1 Department of Chemical Technology, Faculty of Chemistry, Adam Mickiewicz University, Uniwersytetu Poznańskiego 8, 61-614 Poznan, Poland; agata.jankowska@amu.edu.pl; 2 Department of Construction Materials Engineering and Geoengineering, Faculty of Civil Engineering and Architecture, Lublin University of Technology, Nadbystrzycka 40, 20-618 Lublin, Poland; r.panek@pollub.pl (R.P.); w.franus@pollub.pl (W.F.)

**Keywords:** pesticides, water contamination, waste materials, porous adsorbents, adsorption kinetics, mechanism analysis

## Abstract

Global development has led to the generation of substantial levels of hazardous contaminants, including pesticides, which pose significant environmental risks. Effective elimination of these pollutants is essential, and innovative materials and techniques offer promising solutions. This study examines the modification of natural zeolite (clinoptilolite) and fly ash-based NaA and NaX zeolites with hexadecyltrimethylammonium bromide (CTAB) to create inexpensive adsorbents for removing 2,4-dichlorophenoxyacetic acid (2,4-D) herbicide from water. Detailed characterization of these materials was performed, along with an evaluation of the effects of pH, contact time, temperature, and initial 2,4-D concentration on their sorption capacities. The modified samples exhibited significant changes in elemental composition (e.g., reduced SiO_2_ and Al_2_O_3_ content, presence of Br) and textural properties. The adsorption of the pesticide was found to be an exothermic, spontaneous process of pseudo-second-order kinetics and was consistent with the Langmuir model. The highest sorption capacities were observed for samples modified with 0.05 mol L^−1^ CTAB, particularly for Cli_CTAB-0.05_.

## 1. Introduction

The widespread use of pesticides makes them one of the most commonly detected contaminants in the environment. Although pesticides are mainly associated with agriculture, they can also be found in mosquito, flea, and tick repellents and are employed as fungicidal agents in paints, papers, swimming pools, and dry cleaners [1,2]. Their application offers several benefits, e.g., they serve as an effective weapon against pests endangering crops, control invasive species, and ensure the quality of stored agricultural products [3]. However, there are significant downsides to their use. The over-exploitation of synthetic pesticides has led to resistance in target organisms, potentially resulting in the emergence of more threatening populations. Pesticides also accumulate in human tissues, causing numerous health issues, e.g., cancer, allergies, and hormonal disruptions. Continuous pesticide use degrades soil quality, reduces biodiversity, disrupts growth processes, and contaminates the food chain, harming beneficial microorganisms, algae, aquatic invertebrates, fish, plants, wildlife, as well as groundwater, drinking water, and other water resources [4,5,6].

**Figure 1:**
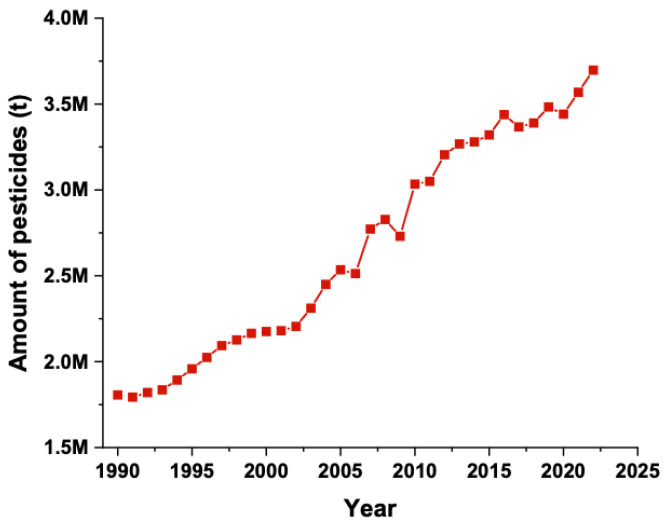
Global use of pesticides since 1990 (Source: FAOSTAT Database) [11].

Fewer than 0.1% of pesticides reach target organisms, such as insects, weeds, and fungi, while the remainder enters surrounding ecosystems, where they can persist for many years [7]. Over recent decades, global usage of these chemicals has increased twofold (Figure 1), contributing to their presence in aquatic environments primarily through direct runoff, leaching, transport processes, improper disposal of empty containers, and equipment washing [8,9]. According to Maximize Market Research, the global pesticide market was valued at US$36.4 billion (bn) in 2023, with Brazil (US$3.75 bn), France (US$2.01 bn), and the United States (US$1.58 bn) as the top importers, and China (US$5.25 bn), France (US$4 bn), and India (US$2.63 bn) as the top exporters [10].

2,4-Dichlorophenoxyacetic acid (2,4-D) is one of the most commonly used pesticides due to its selectivity, high solubility in water and organic solvents, low cost, and effectiveness in controlling a wide range of weeds in crops (e.g., corn, cocoa, coffee, sugar cane). It belongs to the group of herbicides and is regarded as a persistent environmental contaminant as its half-life ranges from 20 to 312 days. It can be applied either directly to the soil or sprayed on plantations, which facilitates its entry into surface and groundwater, resulting in severe ecological impacts, including plants and fish mortality, drinking water contamination, and bioaccumulation. The presence of 2,4-D in water bodies also poses significant health risks to humans and animals due to its mutagenic and toxic properties, contributing to congenital, respiratory, and kidney diseases, and disrupted urinary and endocrine functions [6,12,13].

Addressing pesticide contamination in water resources requires improving and developing treatment techniques to remove these pollutants. Chlorination [14,15,16], reverse osmosis [17,18,19], and advanced oxidation processes [20,21,22] can be used; however, these methods show limited effectiveness against numerous compounds, can be expensive, and require high energy inputs. Adsorption is the most widely used method of water purification due to its simplicity, effectiveness, and low cost [23]. Additionally, numerous high-performance adsorbents are available, including metal–organic frameworks [24,25,26], activated carbons [27,28,29], and zeolites [30,31,32]. These can be synthesized from various waste materials (e.g., fly ash) derived from households, agriculture, and the power industry [33].

Among the various adsorbents applied in the removal of pesticides, zeolites (e.g., clinoptilolite, NaA, NaX) are highly regarded for their biocompatibility, nontoxicity, low cost, high cation-exchange capacity, internal pores, and versatile surface modification options [34,35]. The structure of clinoptilolite (with an HEU-type framework) is characterized by a sheetlike organization into layers perpendicular to the *b* axis. The 2D channel system consists of ten- and eight-membered tetrahedral rings—the larger A and smaller B channels run parallel to the *c* axis intersecting the C channel along the *a* axis. These channels contain exchangeable cations (e.g., Na^+^, K^+^, Ca^2+^) coordinated by water molecules. Four extra-framework cation positions (M(1)–M(4)) are identified, with specific positions for sodium, potassium, calcium, and magnesium ions based on their coordination environment [36]. NaA zeolite (Linde Type A) has a cubic structure with sodalite cages (principal building units) connected by four-membered rings which form a 3D pore network. The central cavities (diameter 11.4 Å) of the cages are interconnected by eight-ring openings (diameter 4.1 Å) creating an open framework [37,38]. The Faujasite framework of NaX consists of 24 tetrahedra cuboctahedral units, known as sodalite cages, which are arranged similarly to carbon atoms in a diamond structure. They are linked through hexagonal prisms (double six-membered rings), generating a 3D porous channel. This structure exhibits 12-membered oxygen ring windows of 8 Å diameter and supercages of 12 Å diameter [39]. Many studies have explored the role of zeolites in treating water for pesticide contamination. Some of them highlight the importance of employing naturally occurring zeolites or those derived from waste materials. For instance, Doan et al. [40] utilized rice husk to solvothermally synthesize bio-zeolite, which was subsequently modified with 10 mM hexadecyltrimethylammonium bromide (CTAB). The material was applied in the elimination of glyphosate and showed a high adsorption capacity of 324 mg g^−1^. Alvarez-García et al. [41] presented research regarding the adsorption effectiveness of methamidophos on clinoptilolite treated with NaCl solution and modified with CTAB at various concentrations. The best result (1.13 mg g^−1^) was obtained with the use of 25 mmol L^−1^ CTAB. Haghjoo et al. [42] reported the synthesis of fly ash-based NaA zeolite functionalized with cationic surfactant CTAB, which exhibited excellent adsorption capacity and glyphosate removal efficiency of 769.23 mg g^−1^ and 98.92%, respectively. Phouthavong et al. [43] established that non-magnetic zeolite beta and magnetic zeolite beta/Fe_3_O_4_ composite from rice husk ash-SiO_2_ could effectively adsorb paraquat, with respective uptakes of 70 mg g^−1^ and 50 mg g^−1^. Recent research has targeted the removal of 2,4-D using surfactant-modified zeolites. Straioto et al. [44] found that natural zeolite clinoptilolite modified with CTAB adsorbed 2,4-D with a capacity of 8 mg g^−1^, influenced by solution pH, temperature, length of process, and initial 2,4-D concentration. Andrunik et al. [45] described the synthesis of fly ash-based zeolites of A and X type and their composites with carbon, followed by their modification with cationic (CTAB) and nonionic (Triton X-100) surfactants. The uptake of 2,4-D for all materials was in the range of ~0.2–1.4 mg g^−1^. A further study by Andrunik et al. [46] focused on CTAB- and Trixton X-100-modified zeolite X and X-C composites and involved estimating the impact of pH, contact time, and initial 2,4-D concentration, yielding sorption capacities from ~0.3 to ~1.8 mg g^−1^.

Given this background, we aimed to perform detailed comparative research on the potential of low-cost, fly ash-based adsorbents synthesized via the hydrothermal method (NaA and NaX zeolite) and naturally occurring clinoptilolite in the removal of 2,4-D from water. The materials were subjected to modifications with CTAB solutions at two different concentrations to thoroughly describe the effect of the modifier on adsorption performance. To the best of our knowledge, while the previous studies have shown the effectiveness of surfactant-modified zeolites in herbicide removal, a direct comparison between these low-cost, fly ash-based zeolites and natural zeolites remains unexplored. Our study also examined how various parameters, including solution pH, the temperature of the process, contact time between adsorbate and adsorbent, and initial solution concentration affect the sorption capacities of the samples. This enabled the selection of the most favorable conditions for the adsorption of the analyzed pesticide. By comprehensively investigating multiple parameters, we address current research gaps, as prior studies have considered only a limited number of variables. While numerous materials have been explored for the removal of 2,4-D from aqueous solutions, there is still a need for adsorbents with enhanced sorption capacities to improve the effectiveness of the process. Our research aims to develop materials with improved performance, addressing limitations observed in existing approaches, such as low adsorption capacity, structural changes upon modification, and limited stability.

## 2. Results and Discussion

### 2.1. Physicochemical Characterization of Adsorbents

The following section provides a detailed physicochemical characterization of the analyzed materials. The results obtained with the use of energy-dispersive X-ray fluorescence (XRF) indicated that the unmodified zeolites are predominantly composed of silica (38.17–68.82%) and alumina (9.57–25.23%) (Table 1). They also consist of smaller amounts of oxides such as CaO (3.50–6.60%), Fe_2_O_3_ (2.30–9.48%), and Na_2_O (4.68–4.77%). The composition of clinoptilolite (Cli), NaA, and NaX contrasts with that of modified adsorbents. Notably, there was a substantial decrease in SiO_2_ (7.90–44.73%) and Al_2_O_3_ (0.98–15.46%) content. The absence or reduced sodium content indicated that CTAB was incorporated into the structure of the samples through a cation exchange mechanism involving Na^+^ and the quaternary ammonium cation. The effectiveness of the modifications was confirmed by the presence of Br at levels of 5.10–9.05% and 16.49–19.68% for CTAB concentrations of 0.05 mol L^−1^ and 0.1 mol L^−1^, respectively. The Na_2_O and Fe_2_O_3_ contents were higher in NaA and NaX, since NaOH was the primary substrate used in their synthesis, and fly ash contains a variety of inorganic oxides, including Fe_2_O_3_. The Si/Al molar ratio is a significant parameter that is often linked to the specific type of zeolite. For the studied samples, these values were as follows: Cli (6.10), Cli_CTAB-0.05_ (4.76), Cli_CTAB-0.1_ (15.96), NaA (1.59), NaA_CTAB-0.05_ (1.59), NaA_CTAB-0.1_ (1.62), and NaX (1.28), NaX_CTAB-0.05_ (1.38), NaX_CTAB-0.1_ (1.43). These values align well with the literature data. The silica-to-alumina ratio changes more sharply for natural clinoptilolite than for the fly ash-based NaA and NaX zeolites upon modification. This difference may be attributed to the higher cation exchange capacity of clinoptilolite, due to its natural origin and structural properties. Clinoptilolite possesses more cation exchange sites, making it more susceptible to interaction and functionalization with CTAB. This can lead to more significant changes in surface composition and, consequently, in the silica-to-alumina ratio. In contrast, the synthetic zeolites (NaA and NaX) may have fewer exchange sites, resulting in more stable silica-to-alumina ratios. The loss of ignition (LOI) was the highest for NaX, owing to the substantial amount of adsorbed water within its structure that is released at temperatures exceeding 100 °C [47,48,49,50,51].

**Figure 2:**
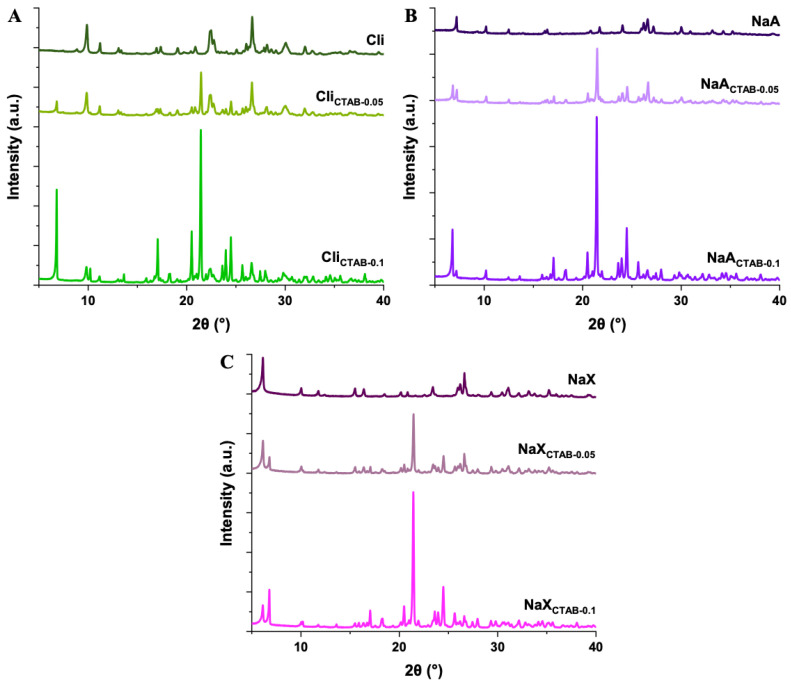
XRD patterns of Cli (**A**), NaA (**B**), NaX (**C**), pure and modified with CTAB, in the angle range 2θ = 5–40°.

Characteristic reflections for all zeolites were identified with X-ray diffraction (XRD) (Figure 2). For Cli the reflections were found, among others, at 2θ ≈ 9.88°, 11.21°, 22.49°, 30.07°, 32.01° (d_hkl_ = 8.94, 7.89, 3.95, 2.97, 2.79 Å). Reflections attributed to quartz, which is considered an impurity, were also visible at 2θ ≈ 20.88°, 26.65°, 36.57°, 39.50° (d_hkl_ = 4.25, 3.34, 2.45, 2.28 Å) [52]. In the case of NaA and NaX, reflections from the zeolite phase were present at 2θ ≈ 7.20°, 10.21°, 24.05°, 27.18°, 30.01° (d_hkl_ = 12.26, 8.65, 3.70, 3.28, 2.97 Å) and 2θ ≈ 6.14°, 10.04°, 15.51°, 26.63°, 31.08° (d_hkl_ = 14.38, 8.80, 5.71, 3.34, 2.87 Å), respectively. Admixtures of mullite or quartz and a higher background level were also observed as the fly ash was not completely converted into zeolite [47,50]. The XRD results of studied zeolites are consistent with the diffractograms of commercially available ones, which proves that they can be successfully synthesized with the use of waste materials. No notable shifts in the position of intense reflections for Cli, NaA, or NaX were detected after modifications, which indicates that CTAB did not alter the structure of materials. New reflections from CTAB, e.g., at 2θ ≈ 6.81°, 21.46°, 24.51°; d_hkl_ = 12.96, 4.14, 3.63 Å (Cli_CTAB-0_._05_), 2θ ≈ 6.81°, 10.22°, 21.44°, 24.49°; d_hkl_ = 12.96, 8.65, 4.14, 3.63 Å (Cli_CTAB-0.1_), 2θ ≈ 6.83°, 21.48°, 24.52°; d_hkl_ = 12.93, 4.13, 3.63 Å (NaA_CTAB-0_._05_), 2θ ≈ 6.79°, 21.43°, 24.48°; d_hkl_ = 13.00, 4.14, 3.63 Å (NaA_CTAB-0.1_), 2θ ≈ 6.80°, 21.45°, 24.51°; d_hkl_ = 12.98, 4.14, 3.63 Å (NaX_CTAB-0_._05_), and 2θ ≈ 6.79°, 21.43°, 24.48°; d_hkl_ = 13.00, 4.14, 3.63 Å (NaX_CTAB-0.1_) could be seen as well. In the case of the Cli_CTAB-0.1_ sample, the observed splitting of the reflection corresponding to the zeolite phase at 9.88° was the result of a new reflection at 10.22° due to modification with CTAB. The intensity of these reflections was higher for a CTAB concentration of 0.1 mol L^−1^ [53,54].

**Figure 3:**
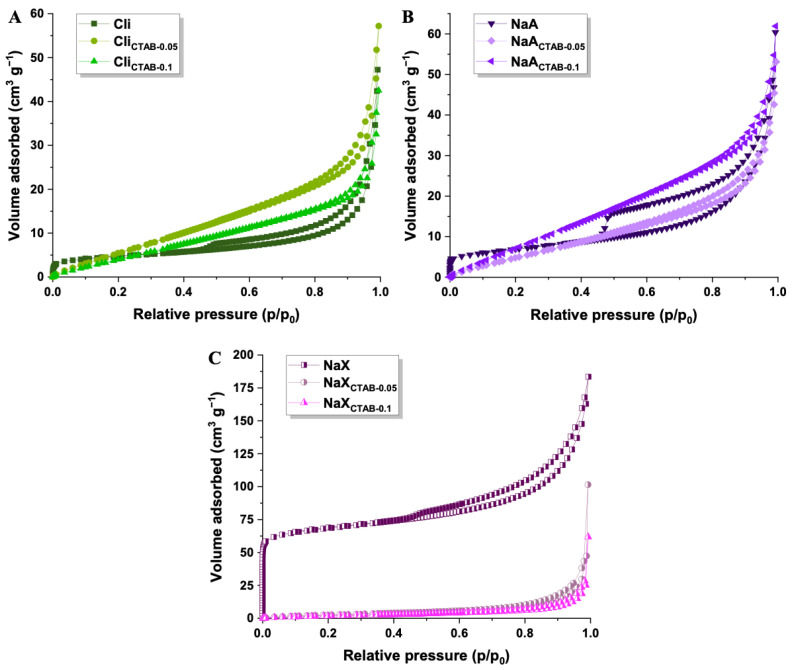
Nitrogen adsorption/desorption isotherms of Cli, Cli_CTAB-0.05_, Cli_CTAB-0.1_ (**A**), NaA, NaA_CTAB-0.05_, NaA_CTAB-0.1_ (**B**), and NaX, NaX_CTAB-0.05_, NaX_CTAB-0.1_ (**C**).

The shape of low-temperature nitrogen adsorption/desorption isotherms for the obtained samples corresponded to a type II isotherm with an H3 type of hysteresis loop, based on classification by the International Union of Applied Chemistry (Figure 3). The H3 hysteresis is commonly observed for materials that possess slit-like pores and is connected to the presence of secondary mesopores (intraparticle mesoporosity) [55,56,57]. A notable rise in nitrogen adsorption at low relative pressure values for NaX denotes a significant contribution of micropores. After the modification of NaX, a substantial drop of adsorbed nitrogen was observed, particularly for the higher concentration of surfactant, and the hysteresis loop was almost negligible as the pores were filled by a modifier, causing a reduction in pore volume. The isotherm shape of Cli_CTAB-0.05_, Cli_CTAB-0.1_, NaA_CTAB-0.05_, and NaA_CTAB-0.1_ also showed changes upon CTAB modification. At low p/p_0_, a reduction in the nitrogen uptake was noticed, which indicated the disappearance of micropores as a result of their blocking by surfactant molecules. The flattening of the desorption branch, characteristic of mesoporous structure, suggested alterations in the shape and size of secondary mesopores. These observed changes are consistent with previous reports on the effects of surfactant modification on pore structure and accessibility, where CTAB has been shown to induce a shift in pore size distribution, lowering micropore volume while facilitating mesopore accessibility [45,58,59,60,61,62].

Table 2 summarizes the textural parameters of all samples. NaX has a higher specific surface area (260 m^2^ g^−1^) as well as pore volume (0.19 cm^3^ g^−1^) than Cli (17 m^2^ g^−1^, 0.03 cm^3^ g^−1^) and NaA (23 cm^3^ g^−1^, 0.05 cm^3^ g^−1^). In addition to mesopores, NaX possesses micropores whose area and volume are 191 m^2^ g^−1^, 0.08 cm^3^ g^−1^. After modification with CTAB, the specific surface area and pore volume decreased in the case of NaX as a result of surfactant molecules present on the surface and within the pores of materials [45]. The t-plot analysis revealed that CTAB molecules blocked micropores and contributed to the larger average pore diameter of NaX_CTAB-0.05_ and NaX_CTAB-0.1_ [63]. For Cli and NaA, the higher specific surface area was observed after modification. The surfactant molecules can create a more uniform surface coverage, which leads to the dispersion of zeolite particles, preventing aggregation and, consequently, increasing specific surface area [64,65,66].

**Figure 4:**
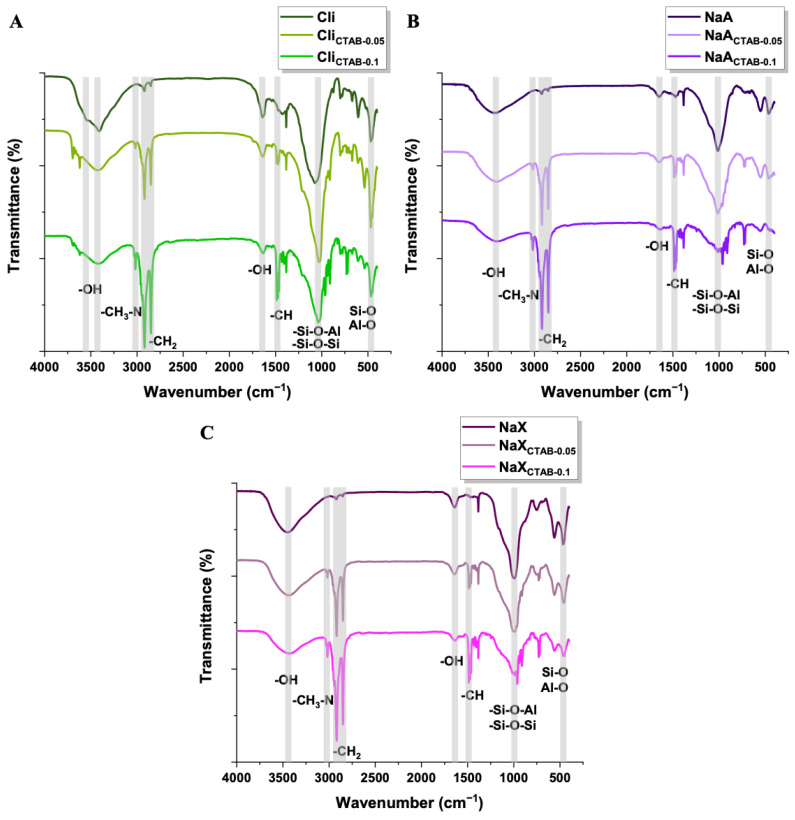
FT-IR spectra of pure and modified Cli (**A**), NaA (**B**), NaX (**C**).

The Fourier transform infrared (FT-IR) spectra of all studied samples displayed characteristic bands at ~3400 cm^−1^ and 1630 cm^−1^, corresponding to the stretching vibrations of structural hydroxyl groups and the bending vibrations of adsorbed water molecules, respectively (Figure 4).

Additionally, for clinoptilolite, the wide band correlates with asymmetric (~3600 cm^−1^) and symmetric (~3400 cm^−1^) vibrations of –OH (Figure 4A). A strong band near 1000 cm^−1^ is indicative of a well-defined aluminosilicate framework, specifically the asymmetric stretching vibrations of Si–O–Si and Si–O–Al bonds. The pseudo-lattice vibrations detected in the range of ~800–500 cm^−1^ are associated with over-tetrahedral structural units—[SiO_4_] and [AlO_4_]. Internal vibrations of Si–O and Al–O bending are identified at ~450 cm^−1^. In the spectra of modified adsorbents, the CTAB vibration bands can derive from alkylammonium head groups and methylene tails. The band at ~3015 cm^−1^ indicates the symmetric stretching vibration of CH_3_–N. The bands around 2900 cm^−1^ and 2850 cm^−1^ are the result of asymmetric and symmetric vibrations of –CH_2_, respectively. Bands at ~1500 cm^−1^ are attributed to the methylene scissoring mode. Their presence implies that the modification with the surfactant has been conducted successfully. The intensity of all the analyzed bands characteristic for CTAB rises with the increase of surfactant concentration used for modification [47,58,67,68].

**Figure 5:**
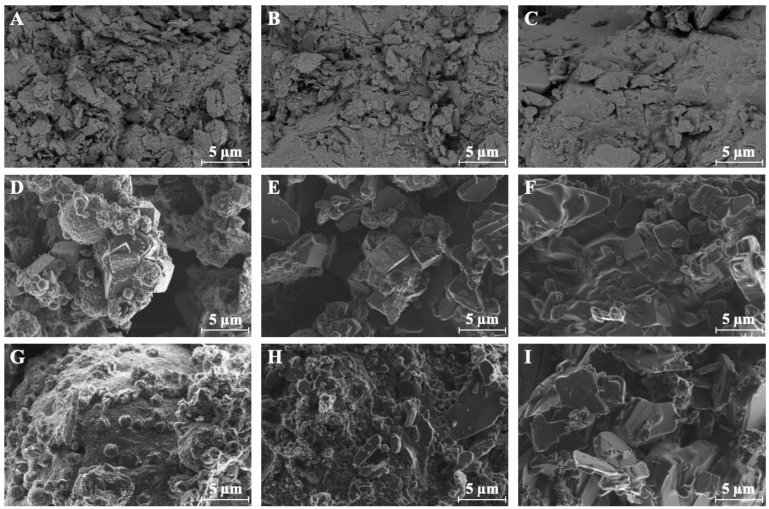
SEM images of Cli (**A**), Cli_CTAB-0.05_ (**B**), Cli_CTAB-0.1_ (**C**), NaA (**D**), NaA_CTAB-0.05_ (**E**), NaA_CTAB-0.1_ (**F**), NaX (**G**), NaX_CTAB-0.05_ (**H**), NaX_CTAB-0.1_ (**I**).

Figure 5 depicts the SEM images of all analyzed samples. The surface of clinoptilolite is rough and its morphology can be labeled as dense agglomerates of different shapes (Figure 5A). Regarding NaA and NaX, the zeolite crystals are formed on spherical or irregularly shaped residual ash or unreacted fragments of aluminosilicate glaze and resemble single crystals or interconnected aggregates (Figure 5D,G). The LTA-type (Linde Type A) zeolite crystallites of NaA are cubic and well-formed. The NaX crystals possess an isomeric structure, exhibit a Faujasite-type framework, and are irregular and sharp-edged.

In the case of Cli_CTAB-0.05_ and Cli_CTAB-0.1,_ it is coated with an organic layer of CTAB which hinders the visualization of structure, pores, and cavities (Figure 5B,C). After modification, the sharpness of NaA and NaX crystal ends was reduced, implying that CTAB molecules created a more even surface on the zeolite crystals (Figure 5E,F,H,I). The observed increase in specific surface area for CTAB-modified Cli and NaA samples, as determined from nitrogen adsorption/desorption isotherms, can be attributed to the surfactant-induced prevention of particle aggregation, which is consistent with the more dispersed particle arrangement visible in SEM images. This indicates that while the organic coating conceals the pore structure, it does not block the accessible surface, contributing to the enhancement in specific surface area [44]. For NaX, the smoother crystal surfaces seen in SEM images are linked to the reduction in specific surface area and pore volume. This is due to the CTAB molecules blocking or filling the pores, leading to reduced nitrogen adsorption as detected by the nitrogen sorption isotherms. Despite these morphological and surface changes, the XRD analysis showed no significant shifts in the position of intense reflections, confirming that the structural integrity of zeolites was maintained [45,47,69,70].

Studies of the particle size distribution for NaX zeolite revealed that the determined parameter d(0.1) was 6.56 µm, indicating that 10% of the particles are smaller than this size (Table 3). Half of the particles are smaller than 15.7 µm, as indicated by d(0.5) = 15.7 µm, while d(0.9) = 33.6 µm means that 90% of the particles are smaller than 33.6 µm, signifying the presence of larger particles within the distribution. The results of the grain composition of NaA zeolite showed that the parameter d(0.1) = 3.21 µm is slightly smaller than for NaX zeolite. This denotes a relatively fine fraction of particles. The parameter d(0.5) was 17.1 µm, reflecting a slightly coarser middle part compared to the previous sample. The presence of a larger fraction of coarse particles was suggested by d(0.9) = 67.8 µm. For clinoptilolite, the parameter d(0.1) was 3.40 µm, indicating the presence of fine particles in this sample, while d(0.5) was 26.7 µm, which showed a thicker middle section compared to synthetic zeolites. The parameter d(0.9) = 97.8 µm signified a wider particle size distribution and the presence of larger particles in the material. The results of particle size distribution for the studied materials are consistent with the findings from the SEM analysis [71].

### 2.2. Adsorption of 2,4-D

#### 2.2.1. pH Impact

**Figure 6:**
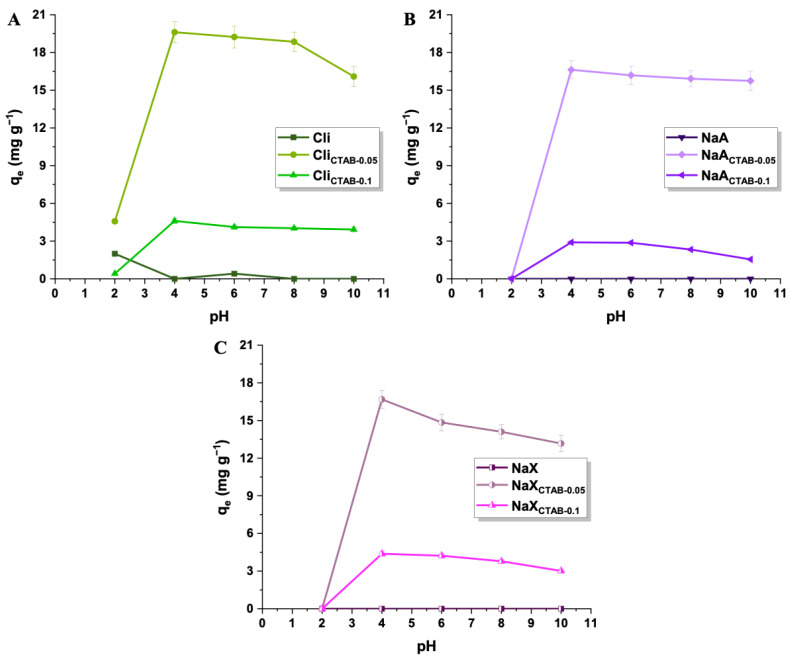
Effect of pH (2–10) on the removal of pesticide with the use of pure and CTAB-modified Cli (**A**), NaA (**B**), and NaX (**C**) (2,4-D concentration: 25 mg L^−1^, adsorbent mass: 50 mg).

The point of zero charge (pzc) for each sample provides understanding of how adsorbents interact with 2,4-D at different pH levels of the solution. The values of this parameter for the studied materials are as follows: Cli (6.60), Cli_CTAB-0.05_ (5.36), Cli_CTAB-0.1_ (6.03), NaA (10.51), NaA_CTAB-0.05_ (10.11), NaA_CTAB-0.1_ (10.02), and NaX (10.26), NaX_CTAB-0.05_ (9.90), NaX_CTAB-0.1_ (9.84). In the case of all CTAB-modified materials, pH 4 proved the most favorable environment for 2,4-D adsorption, as the charges on the surface of samples and pesticide molecules enable electrostatic interactions (Figure 6). The pesticide exists in the anionic form at a pH higher than its pK_a_ and, based on the literature data, the value of pK_a_ for 2,4-D is 2.8 [72]. The surface of materials is positively charged at a pH lower than their experimentally determined pzc. Therefore, electrostatic attraction between adsorbents and 2,4-D occurred at a pH value of 4 for all samples, leading to the most effective removal of 2,4-D [73]. With increasing pH, the uptake of the pesticide slightly decreased due to the repulsion between negatively charged adsorbent and adsorbate molecules or reduction in the zeolite’s positive surface charge [48]. At lower pH levels, the sorption capacities dropped significantly (0–5 mg g^−1^), since protons in a highly acidic solution initiate the hydrolysis of Si–O–Al, causing dealumination and splitting of the bonds in the zeolite structure [74,75].

#### 2.2.2. Contact Time Impact

**Figure 7:**
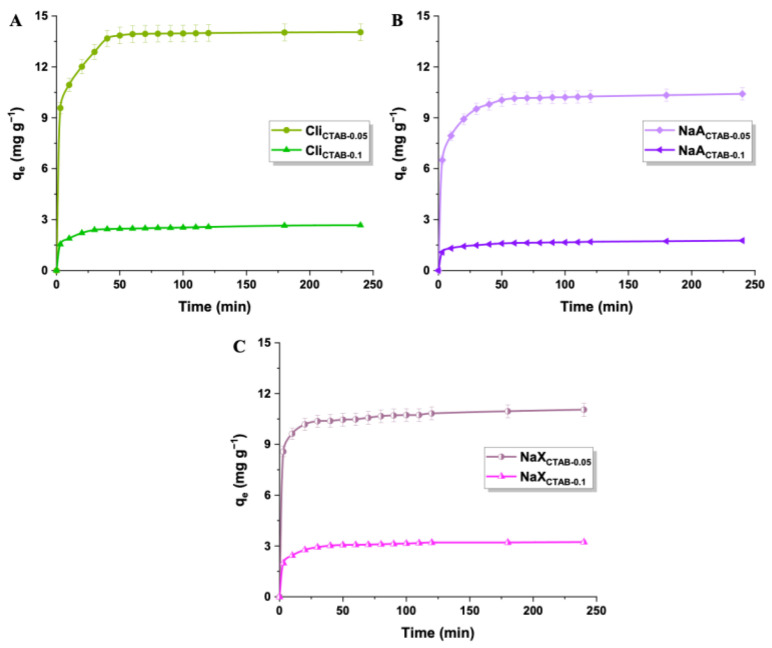
Impact of contact time (0–240 min) on the adsorption of 2,4-D onto Cli (**A**), NaA (**B**), and NaX (**C**) modified with CTAB (2,4-D concentration: 15 mg L^−1^, adsorbent mass: 50 mg).

The adsorption of the herbicide onto CTAB-modified samples occurred in two stages, an initial rapid phase within the first 20 min and a more gradual phase lasting until equilibrium was attained (Figure 7). The rapid stage resulted from the abundance of available active sites on the adsorbent surface, which enhanced mass transfer and 2,4-D molecule capture. Over time, the adsorbents’ pores and active sites became occupied. Herbicide molecules (10.5 Å × 7.5 Å × 5 Å [76]) moved deeper into the pores and encountered more resistance, which caused a reduction in the adsorption speed until the state of equilibrium was reached [73,77,78].

#### 2.2.3. Kinetic Studies

The data displayed in Table 4 indicate that the pseudo-second-order model (R^2^ = 0.9993–0.9999) provided a better fit for the adsorption kinetics of 2,4-D adsorption onto CTAB-modified materials compared to the pseudo-first-order model (R^2^ = 0.8404–0.9406).

The experimentally determined amounts of adsorbed 2,4-D (q_e(exp)_) were closer to the q_e_ values for the pseudo-second-order model [73]. This implies that adsorbate concentration on the surface of zeolites is the key factor that affects the adsorption rate, emphasizing the role of surface chemistry and the number of available adsorption sites [79]. The intra-particle diffusion model was applied to fit the data as well. Since the fitting curve does not intersect the origin, it can be suggested that it was not the only rate-limiting step in the adsorption process. The initial phase is characterized by the quick adsorption of 2,4-D on the surface of materials. During the second stage, adsorption occurs gradually with the rate being limited by pore and intra-particle diffusion [73,80].

#### 2.2.4. Temperature Impact

**Figure 8:**
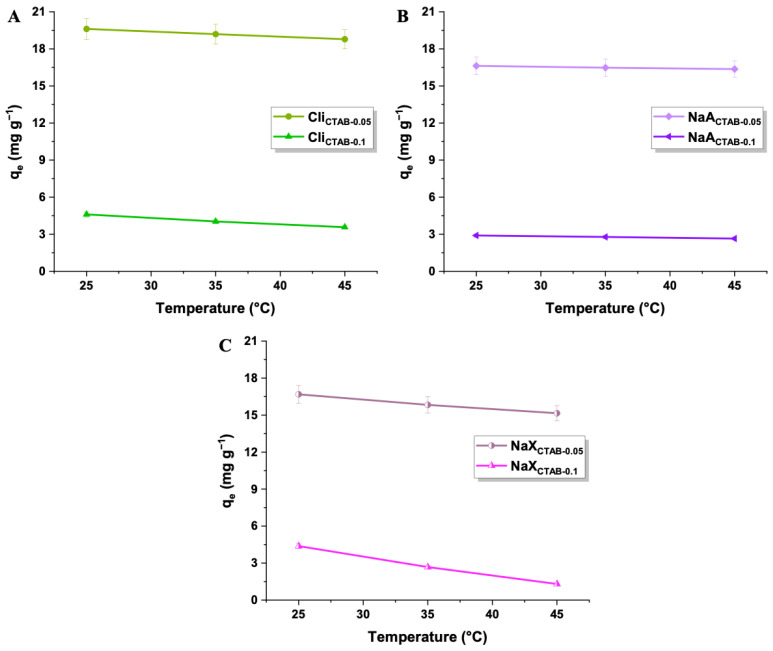
Temperature influence (RT, 35, 45 °C) on 2,4-D uptake on Cli_CTAB-0.05_, Cli_CTAB-0.1_ (**A**), NaA_CTAB-0.05_, NaA_CTAB-0.1_ (**B**), and NaX_CTAB-0.05_, NaX_CTAB-0.1_ (**C**) (2,4-D concentration: 25 mg L^−1^, adsorbent mass: 50 mg).

The analysis of the temperature of the process showed that it does not significantly influence the sorption capacities of modified materials. The uptake of 2,4-D was slightly lower at higher temperatures, hence room temperature was chosen as optimal for further experiments (Figure 8). The adsorbent–adsorbate physical interactions were weakened, since 2,4-D molecules moved more rapidly and overcame intermolecular forces [81]. The process was proven to be exothermic by the negative values of ΔH° (Table 5). The positive ΔS° values implied that the adsorption at the solid–liquid interface is random, and a greater degree of randomness was observed for samples modified with 0.05 mol L^−1^ CTAB. ΔG° values were below zero, which evidenced the spontaneity of 2,4-D adsorption and indicated that it was thermodynamically favorable [82,83,84].

#### 2.2.5. Initial Concentration Impact

**Figure 9:**
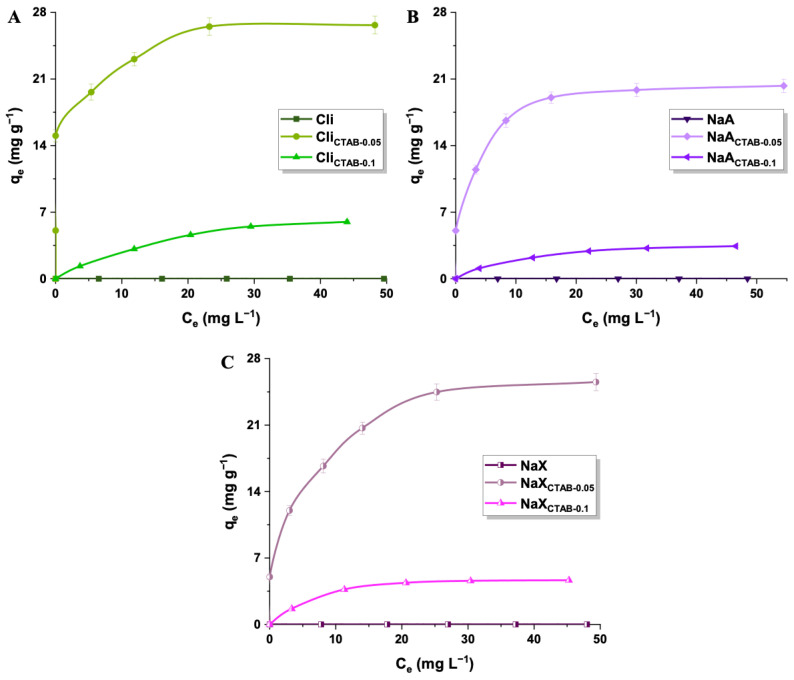
Adsorption of 2,4-D onto pure and modified adsorbents: Cli (**A**), NaA (**B**), and NaX (**C**) (2,4-D concentration: 5–75 mg L^−1^, adsorbent mass: 50 mg).

**Figure 10:**
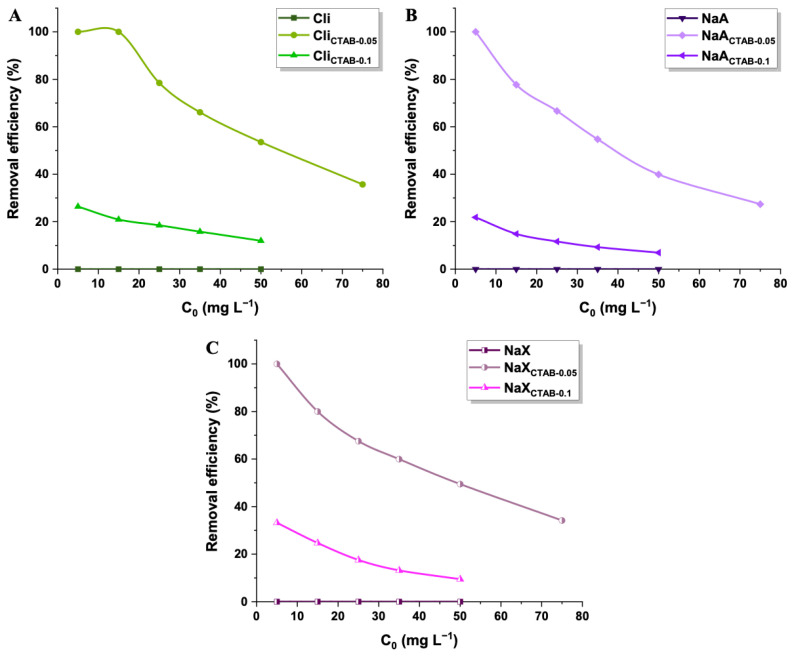
Removal efficiencies of Cli, Cli_CTAB-0.05_, Cli_CTAB-0.1_ (**A**), NaA, NaA_CTAB-0.05_, NaA_CTAB-0.1_ (**B**), NaX, NaX_CTAB-0.05_, and NaX_CTAB-0.1_ (**C**).

**Figure 11:**
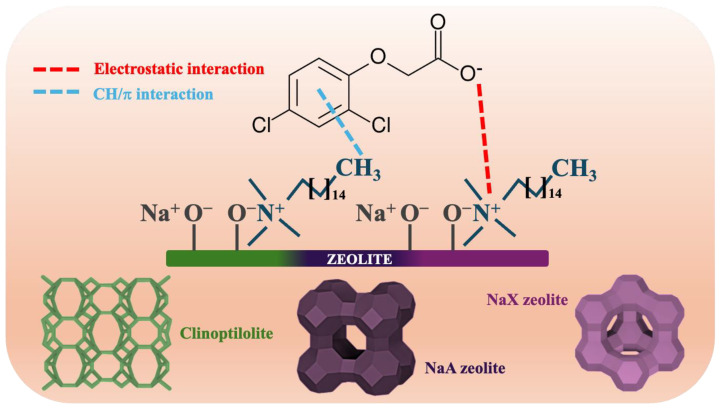
Interactions between analyzed materials and 2,4-D.

The 2,4-D adsorption effectiveness was significantly affected by the initial concentration of herbicide solution (Figure 9). The experiments were conducted at optimal conditions—a pH value of 4 and room temperature. The sorption capacities of synthesized materials increased with higher initial 2,4-D concentration, up to the point of saturation. This effect could be attributed to the stronger driving force that facilitates the mass transfer of pesticide molecules from the bulk solution to the adsorbent surface [85]. The highest removal efficiencies were estimated for the lowest concentration of herbicide, which may be explained on the basis of the adsorbent’s active sites saturation (Figure 10). At higher 2,4-D concentrations, the adsorbent’s capacity to bind additional molecules becomes limited, resulting in a lower herbicide removal percentage. The most efficient material was Cli_CTAB-0.05_, which eliminated 100% of 15 mg L^−1^ 2,4-D [86]. It was not possible to remove 2,4-D from water solutions with the use of pure materials, likely due to their hydrophilic surfaces, which allowed water molecules to occupy adsorption sites, preventing 2,4-D from interacting with the surface. The absence of functional groups derived from the surfactant might also limit the electrostatic forces between 2,4-D and adsorbents [87,88,89,90,91]. The sorption capacities were greatly improved after performing modifications with CTAB. The increase in 2,4-D uptake could be primarily attributed to the positively charged head of surfactant N^+^(CH_3_)_3_ on the outer surface of the samples, which induced electrostatic attraction with negatively charged pesticide molecules (Figure 11). The quaternary ammonium groups introduced by CTAB modification are the primary active sites. These findings are consistent with established pzc and pK_a_ values of adsorbents and 2,4-D [92,93]. For the enhanced 2,4-D removal, the weak hydrogen bond between the C-H group of CTAB and π system of 2,4-D may also be significant [94]. In the case of all studied materials, a CTAB solution concentration of 0.05 mol L^−1^ was more favorable than 0.1 mol L^−1^ (sorption capacities of materials: 20–27 mg g^−1^ and 3–6 mg g^−1^), as higher surfactant concentrations promoted cation exchange within material’s structure, decreasing the specific surface area and hindering the access of the adsorbate to the active sites [95,96]. Although Cli_CTAB-0.05_ displayed the highest sorption capacity (27 mg g^−1^), NaA_CTAB-0.05_ and NaX_CTAB-0.05_ exhibited similar values (20 mg g^−1^ and 26 mg g^−1^). The slightly better result in the case of clinoptilolite may arise from the fact that it possesses more cation exchange sites due to its natural origin, which could make it more applicable for CTAB functionalization. While natural zeolite, such as clinoptilolite, is readily available, synthetic adsorbents such as NaA and NaX show some advantages over natural materials. They can be obtained in a short amount of time, often from waste sources, with the possibility of controlling synthesis parameters that yield an adsorbent with specific properties, high purity, and uniform particle size [97,98].

**Figure 12:**
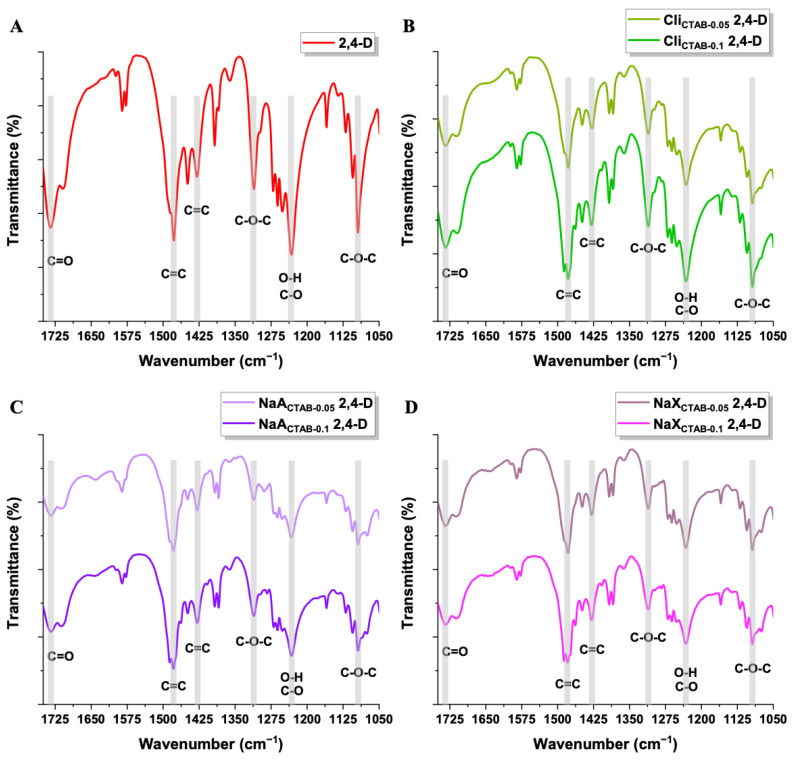
FT-IR spectra of 2,4-D (**A**) and Cli_CTAB-0.05_, Cli_CTAB-0.1_ (**B**), NaA_CTAB-0.05_, NaA_CTAB-0.1_ (**C**), NaX_CTAB-0.05_, NaX_CTAB-0.1_ (**D**) after 2,4-D adsorption.

In the FT-IR spectra of samples after the adsorption process, bands related to the presence of 2,4-D can be found in the range of 1750–1050 cm^−1^, which indicates the effective adsorption of the pesticide on the surface of materials (Figure 12). Two bands at ~1480 cm^−1^ and ~1425 cm^−1^ suggest C=C aromatic ring vibrations. Antisymmetric and symmetric vibrations detected at ~1315 cm^−1^ and ~1090 cm^−1^ belong to C–O–C bond. The bands at around 1735 cm^−1^ and 1230 cm^−1^ are attributed to the carboxylic group (C=O bond and O–H deformation overlapping with the C–O stretching vibration, respectively). Small shifts in the position of the bands compared to the spectrum of 2,4-D suggest the presence of interactions between the studied adsorbents and adsorbate [99,100].

Table 6 presents a comparison of the sorption capacities for 2,4-D among the studies materials (Cli_CTAB-0.05_ and NaX_CTAB-0.05_) and other previously reported adsorbents. The studied samples turned out to be more effective than CTAB-modified (at concentrations equivalent to 1.0 exchange cation-exchange capacity of each material) fly ash-based zeolite X (~0.4 mg g^−1^ [45], ~0.6 mg g^−1^ [46]), zeolite A and carbon composite (~0.6 mg g^−1^ [45]), zeolite A (~0.65 mg g^−1^ [45]), zeolite X and carbon composite (~1.4 mg g^−1^ [45], ~1.8 mg g^−1^ [46]), Fe_3_O_4_ magnetic nanoparticles modified with CTAB (4.9 mg g^−1^ [101]), natural zeolite after CTAB modification (8 mg g^−1^ [44]), fish scale-derived carbon/apatite composite (11.1 mg g^−1^ [102]), or raw sterile bract of *Araucaria angustifolia* (18.95 mg g^−1^ [103]). A higher pesticide uptake was observed in the case of cobalt-grafted activated carbon from date pits and stems (31 mg g^−1^ [104]), amino-functionalized poly (glycidyl methacrylate) (99.45 mg g^−1^ [105]), aminosilane-grafted mesoporous carbon (191 mg g^−1^ [106]), and quaternary amine anionic-exchange MOF UiO-66 (279 mg g^−1^ [107]). It may be a result of different textural parameters, such as higher specific surface area (e.g., 71 m^2^ g^−1^ for aminosilane-grafted mesoporous carbon and 484 m^2^ g^−1^ for quaternary amine anionic-exchange MOF UiO-66). The precise comparison of efficiency of various adsorbents in 2,4-D removal is made difficult by the diversity of experimental conditions, such as pH, under which these studies were conducted [108].

#### 2.2.6. Adsorption Isotherm Models

**Figure 13:**
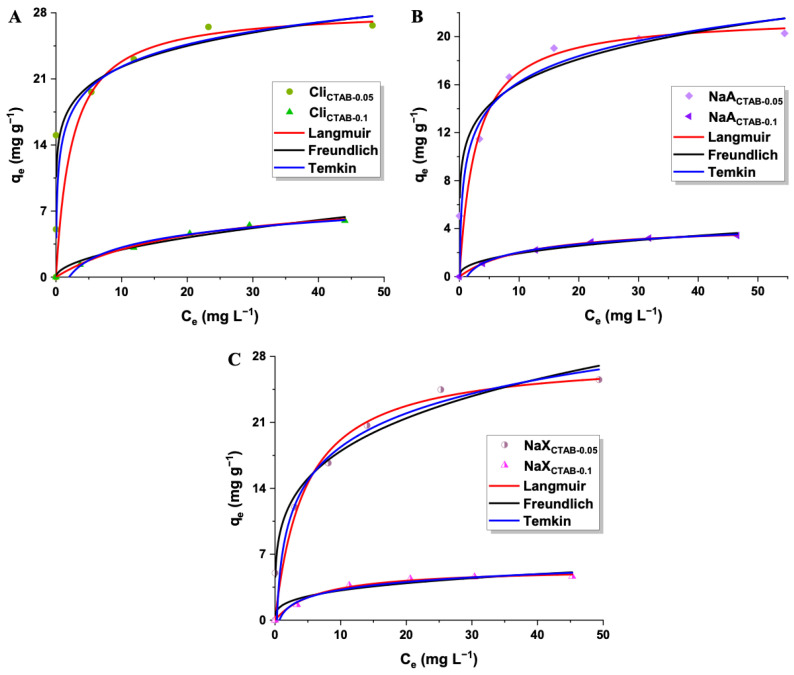
Non-linear fitting of pesticide adsorption isotherms to Langmuir, Freundlich, and Temkin models for Cli_CTAB-0.05_, Cli_CTAB-0.1_ (**A**), NaA_CTAB-0.05_, NaA_CTAB-0.1_ (**B**), NaX_CTAB-0.05_, NaX_CTAB-0.1_ (**C**).

Fitting the obtained data into non-linear Langmuir, Freundlich, and Temkin models showed that 2,4-D adsorption on each material, with the exception of Cli_CTAB-0.05_, was better described by the first model, as the values of R^2^ were higher (Figure 13, Table 7). This implies that adsorption occurs in a single layer on evenly spread active sites on the adsorbent’s surface. The calculated maximum adsorption capacities (q_max_) closely matched the experimental values (q_e(exp)_) [106]. The materials modified with 0.05 mol L^−1^ CTAB exhibited higher K_L_ values (0.216–0.407 L mg^−1^) in comparison with 0.1 mol L^−1^ CTAB (0.048–0.154 L mg^−1^), which denotes their stronger affinity to 2,4-D. The 1/n values computed from the Freundlich model ranged from 0 to 1, suggesting that the adsorption process for the pesticide was energetically favorable [109]. The b_T_ Temkin constant related to the heat of adsorption was in the range of 0.480–2.541 kJ mol^−1^. This indicates that the adsorption of 2,4-D occurred through physisorption, which is associated with low adsorption energies. The K_T_ constant corresponding to the maximum binding energy was higher for 0.05 mol L^−1^ CTAB-modified materials (3.52–71.16 L g^−1^) than for 0.1 mol L^−1^ CTAB-modified zeolites (0.50–1.49 L g^−1^) [110,111].

## 3. Materials and Methods

### 3.1. Preparation of Samples

A series of hydrothermal reactions were carried out, yielding zeolites of type A and X. The zeolites were obtained from fly ash containing trace amounts of carbon from conventional combustion of hard coal in the Jaworzno Power Plant (Jaworzno, Poland). NaX was synthesized in a 24 h reaction from 10 g of ash with 200 mL of 3 mol L^−1^ aqueous NaOH solution (Stanlab, Lublin, Poland). The process was performed at 70 °C without stirring. NaA was obtained in a shorter time (6 h) at a higher temperature of 95 °C with the addition of 0.5 g of the aluminum source (aluminum foil). The products were separated, washed with 2 L of distilled water, and dried at 105 °C for 6 h. The content of NaX in the zeolite material was calculated at 82%, while for NaA the amount of the zeolite phase was 85%. Both of these values were calculated using the Rietveld method. The remainder was the unreacted ash consisting mainly of quartz and mullite, and very small amounts of amorphous phase. Natural zeolite clinoptilolite in the form of zeolitic tuff composed of 75% of pure clinoptilolite phase (according to the mine’s data), cristobalite, orthoclase, quartz, feldspar, and clay minerals e.g., montmorillonite and illite, was acquired from Sokyrnytsya deposit (Transcarpathian region, Ukraine).

Clinoptilolite, NaA, and NaX zeolites were modified with 0.05 and 0.1 mol L^−1^ CTAB aqueous solutions (VWR Chemicals, Solon, OH, USA). The materials (5 g) were placed in a glass bottle and 312.5 mL of a respective solution was added. The samples were agitated for 6 h in an IKA 4000 i control incubator shaker (Staufen, Germany), filtered, and left overnight in a laboratory dryer (Memmert UF 110plus, Schwabach, Germany) at 70 °C. Depending on the concentration of the CTAB solution, the products were denoted as: Cli_CTAB-0.05_, Cli_CTAB-0.1_, NaA_CTAB-0.05_, NaA_CTAB-0.1_, NaX_CTAB-0.05_, and NaX_CTAB-0.1_.

### 3.2. Characterization of Adsorbents

The elemental composition of the samples was determined by energy-dispersive X-ray fluorescence on a Panalytical Epsilon 3 spectrometer (Malvern, UK). The study was carried out in the Na-Am range on an instrument equipped with an Rh 9 W, 50 kV, 1 mA X-ray tube, a 4096 channel spectrum analyzer, six measurement filters (Cu-500, Cu 300, Ti, Al-50, Al-200, Ag), and a high-resolution solid-state SDD detector (Be window, 50 µm thick) cooled by a Peltier cell. The obtained data were analyzed with consideration for the results of the standard LOI test.

The phase composition of the studied adsorbents was assessed by X-ray diffraction. The analysis was performed in the angle range of 2θ = 5–40° with a step of 0.01° on a Panalytical X’pert PROMPD diffractometer (Malvern, UK) equipped with a PW 3050/60 goniometer. A copper lamp with CuKα radiation 0.154178 nm served as an X-radiation source.

Estimation of specific surface area, pore volume, and pore size of synthesized materials was realized based on low-temperature (−194.85 °C) nitrogen adsorption/desorption with a 3Flex 3500 specific surface area analyzer (Micromeritics Instrument Corporation, Norcross, GA, USA). The initial step involved degassing all samples at 200 °C for 24 h. The Brunauer–Emmet–Teller (BET) method, over a relative pressure range of 1.5 × 10^−7^ and 0.99, was employed to determine the specific surface area of materials. Total pore volume was calculated based on a single point (p/p0~0.99) on the desorption isotherm and the average pore size was ascertained using the Barret–Joyner–Halenda (BJH) method. The t-plot analysis was used to compute micropore area and volume.

Determination of pzc was conducted through a pH drift method. A sodium chloride solution (POCH S.A., Gliwice, Poland) at a concentration of 0.01 mol L^−1^ was prepared and hydrochloric acid (Stanlab, Lublin, Poland) or sodium hydroxide (Stanlab, Lublin, Poland) were then added to adjust the pH to the values of 2, 4, 6, 8, 10, and 12 with an Aqualytic Portable meter AL10pH (Dortmund, Germany). A quantity of 50 mL of each of these solutions was mixed with 150 mg of each material and shaken at 200 rpm for 24 h. The final pH was measured and the value corresponding to the initial pH was identified as pHpzc.

The Fourier transform infrared spectra were recorded in the wavenumber range of 4000–400 cm^−1^ using a Bruker IFS 66/s spectrometer (Billerica, MA, USA) to identify and compare the surface functional groups of the materials before and after 2,4-D adsorption. The samples were prepared by grounding 0.5 mg of each zeolite with 200 mg of potassium bromide, which mixtures were then formed into tablets for measurement.

The materials’ surface morphologies were analyzed using an ultra-high-resolution analytical focused ion beam scanning electron microscope (FIB-SEM) (Scios 2 LoVac, manufactured by Thermo Fisher Scientific, Waltham, MA, USA) equipped with an energy-dispersive X-ray-based chemical composition analyzer (UltraDry Premium EDS, also from Thermo Fisher Scientific). The microscope featured a Schottky Field Emission Cathode and NICol ultra-high resolution (UHR) electron column, which operated in non-immersion mode. The samples were glued on aluminum holders using carbon tape, and then coated with a layer of carbon approximately 30 nm thick to provide conductivity. The study was performed in high-vacuum mode using a special Thermo Scientific Trinity Detection System, designed for simultaneous angular image registration and energy-selective scanning electron microscopy (SE) and backscatter electron imaging (BSE). This enabled high-resolution observations using an accelerating voltage of 2 kV and a beam current of 25 pA.

The grain size study was carried out by laser diffraction using the Mastersizer 3000 analyzer with HYDRO EV add-on from Malvern Panalytical, Malvern, UK. The measurement was performed in a dispersing liquid (demineralized water). Grains with equivalent diameters in the range of 0.1 to 1000 μm were analyzed according to the Mie theory. The results were described as percentages of d(0.1), d(0.5), d(0.9) defined as follows: d(0.1) (µm)—10% of the particles are smaller than the measured size; d(0.5)—the median where half of the particles have a size above this value and half below; and d(0.9)—90% of particles are smaller than the measured size.

### 3.3. Adsorption Studies

To determine the sorption capacities of adsorbents for 2,4-D, 50 mg of material was mixed with 50 mL of pesticide solution (Sigma-Aldrich, Saint Louis, MO, USA) at concentrations between 5 mg L^−1^ and 75 mL L^−1^, followed by agitating the samples at room temperature overnight. The mixtures were filtered and the measurement of absorbance of collected supernatants was conducted using an Agilent Cary 60 UV-Vis spectrophotometer (Santa Clara, CA, USA). To calculate the amount of 2,4-D adsorbed onto the surface of zeolites and the removal efficiency, Formulas (1) and (2) were used:

(1) $$q_{e}=\frac{(C_{0}-C_{e})\times V}{m}$$

where: q_e_—the amount of adsorbed 2,4-D (mg g^−1^), C_0_—the initial concentration of 2,4-D solution (mg L^−1^), C_e_—the residual concentration of 2,4-D solution (mg L^−1^), V—the volume of 2,4-D solution (L), m—the mass of the sample (g).

(2) $$\%R=\frac{\left(C_{0}-C_{e}\right)}{C_{0}}\times 100$$

where: %R—removal efficiency (%), C_0_—the initial concentration of 2,4-D solution (mg L^−1^), C_e_—the residual concentration of 2,4-D solution (mg L^−1^).

Non-linear Langmuir (Equation (3)), Freundlich (Equation (4)), and Temkin (Equation (5)) isotherm models were employed to explain the adsorption mechanism. It was determined based on R^2^ values. The models are described by the equations:

(3) $$q_{e}=q_{max}K_{L}\frac{C_{e}}{1+K_{L}C_{e}}$$

where: q_e_—the amount of adsorbed 2,4-D (mg g^−1^), q_max_—the theoretical maximum adsorption capacity of the material (mg g^−1^), K_L_—the Langmuir constant (L mg^−1^), C_e_—the residual concentration of 2,4-D solution (mg L^−1^),

(4) $$q_{e}=K_{F}{C_{e}}^{1/n}$$

where: q_e_—the amount of adsorbed 2,4-D (mg g^−1^), K_F_—the Freundlich constant (mg g^−1^ (L mg^−1^)^1/n^), C_e_—the residual concentration of 2,4-D solution (mg L^−1^), n—the Freundlich constant,

(5) $$q_{e}=\frac{RT}{b_{T}}\ln K_{T}C_{e}$$

where: q_e_—the amount of adsorbed 2,4-D (mg g^−1^), R—universal gas constant (8.314 J K^−1^ mol^−1^), T—absolute temperature (K), b_T_—Temkin constant (J mol^−1^), K_T_—Temkin constant (L g^−1^), C_e_—the residual concentration of 2,4-D solution (mg L^−1^).

The influence of pH (2, 4, 6, 8, 10) was investigated by preparing 2,4-D solution in the concentration of 25 mg L^−1^ and adding hydrochloric acid or sodium hydroxide to regulate the pH values. 50 mg of each material was used, and the above-described procedure was subsequently applied to carry out the adsorption process.

In order to assess the impact of contact time between materials (50 mg) and 2,4-D molecules (solution concentration 15 mg L^−1^), the process was performed in the time range of 0–240 min. The characterization of kinetics of 2,4-D adsorption on the obtained samples involved comparing it to the three kinetic models: Lagergren’s pseudo-first-order model (Equation (6)) [112], the pseudo-second-order model by Ho and McKay (Equation (7)) [113], and the intra-particle diffusion model proposed by Weber and Morris (Equation (8)) [114]. The equations that represent these models are as follows:

(6) $$\ln(q_{e}-q_{t})=\ln q_{e}-\frac{k_{1}t}{2.303}$$

where: q_e_—amount of 2,4-D adsorbed at equilibrium (mg g^−1^), q_t_—amount of 2,4-D adsorbed at time t (mg g^−1^), k_1_—the rate constant of Lagergren’s pseudo-first-order kinetic model (min^−1^), t—the time (min),

(7) $$\frac{t}{q_{t}}=\frac{1}{k_{2}{q_{e}}^{2}}+\frac{t}{q_{e}}$$

where: t—the time (min), q_t_—amount of 2,4-D adsorbed at time t (mg g^−1^), k_2_—the rate constant of Ho and McKay pseudo-second-order kinetic model (g mg^−1^ min^−1^), q_e_—amount of 2,4-D adsorbed at equilibrium (mg g^−1^),

(8) $$q_{t}=k_{i}t^{1/2}+C$$

where: q_t_—the amount of 2,4-D adsorbed at time t (mg g^−1^), k_i_—the rate constant of the intra-particle diffusion model (mg g^−1^ min^−1/2^), t—the time (min), and C—the intercept.

To evaluate the temperature effect, the adsorption experiment was carried out at room temperature, 35, and 45 °C using a 25 mg L^−1^ solution of pesticide and 50 mg of adsorbent. The analysis of thermodynamic parameters such as standard Gibbs free energy (ΔG°), standard adsorption enthalpy (ΔH°), and standard entropy (ΔS°) was conducted to demonstrate the energy effects concerning adsorption. The following formula was employed to ascertain ΔG° values:

(9) $$\Delta G{}^{\circ}=-RT\ln K_{L}$$

where: ΔG°—standard Gibbs free energy (kJ mol^−1^), R—universal gas constant (8.314 J K^−1^ mol^−1^), T—absolute temperature (K), K_L_—the Langmuir constant (L mol^−1^).

Based on the ΔG° vs. T plot, the values of ΔH° as well as ΔS° were computed using the equation provided here:

(10) ΔG°=ΔH°−TΔS°

where: ΔG°—standard Gibbs free energy (kJ mol^−1^), ΔH°—standard adsorption enthalpy (kJ mol^−1^), T—absolute temperature (K), ΔS°—standard entropy (J K^−1^ mol^−1^) [115].

## 4. Conclusions

Different concentrations of the cationic surfactant CTAB were used to modify natural and fly ash-based zeolites, which were then applied to remove the pesticide 2,4-D from water through adsorption. The successful modification of the samples was confirmed by changes observed in elemental composition, diffraction patterns, textural parameters, and FT-IR spectra, which indicated the effective incorporation of CTAB into the zeolite structures through the cation exchange process, whereby sodium ions were replaced by quaternary ammonium ions. The low-temperature nitrogen adsorption/desorption isotherms align with type II. SEM images revealed that the presence of CTAB resulted in a more uniform surface of the zeolite crystals. The adsorption of the pesticide is a spontaneous and exothermic process that is well described by the Langmuir model. Modification with CTAB, particularly with 0.05 mol L^−1^ solution, significantly enhanced the sorption capacities of adsorbents. Electrostatic interactions between positively charged N^+^(CH_3_)_3_ groups of the samples and negatively charged 2,4-D molecules at pH 4 promoted the uptake of the pesticide (20–27 mg g^−1^). Cli_CTAB-0.05_ was the most efficient in the removal of 2,4-D, likely due to having the greatest number of cation exchange sites. This study demonstrates that employing modified fly ash-derived materials in water treatment not only promotes the recycling of wastes but also offers a feasible option for water decontamination. Future research should explore the use of these adsorbents for the removal of other persistent organic pollutants from water, to further expand their potential role in environmental remediation.

## Figures and Tables

**Table 1:** Elemental composition of Cli, Cli_CTAB-0.05_, Cli_CTAB-0.1_, NaA, NaA_CTAB-0.05_, NaA_CTAB-0.1_, and NaX, NaX_CTAB-0.05_, NaX_CTAB-0.1_ materials.

Material	Content (%)							
	SiO_2_	Al_2_O_3_	Na_2_O	CaO	Fe_2_O_3_	Br	Other	LOI
Cli	68.82	9.57	-	3.50	2.30	-	4.66	11.15
Cli_CTAB-0.05_	44.73	7.97	-	2.32	1.76	5.10	3.14	34.98
Cli_CTAB-0.1_	18.43	0.98	-	1.00	0.86	16.53	0.27	61.93
NaA	41.89	22.29	4.77	6.60	9.48	0.03	6.89	8.05
NaA_CTAB-0_._05_	28.90	15.46	1.70	5.05	7.25	8.30	4.77	28.57
NaA_CTAB-0.1_	9.51	4.99	-	2.11	3.32	19.68	1.81	58.58
NaX	38.17	25.23	4.68	3.84	8.11	-	5.02	14.95
NaX_CTAB-0_._05_	22.21	13.69	1.49	2.72	5.37	9.05	2.94	42.53
NaX_CTAB-0.1_	7.90	4.69	0.22	1.26	2.57	16.49	1.15	65.72

**Table 2:** Textural parameters obtained for the studied samples.

Material	Specific Surface Area (m^2^ g^−1^)	Total Pore Volume (cm^3^ g^−1^)	Average Pore Diameter (nm)	Micropore Volume (cm^3^ g^−1^)
Cli	17	0.03	19.37	-
Cli_CTAB-0.05_	24	0.04	10.38	-
Cli_CTAB-0.1_	18	0.03	10.56	-
NaA	23	0.05	10.75	-
NaA_CTAB-0.05_	30	0.04	10.83	-
NaA_CTAB-0.1_	50	0.05	8.40	-
NaX	260	0.19	11.66	0.08
NaX_CTAB-0.05_	12	0.03	40.07	-
NaX_CTAB-0.1_	9	0.02	34.93	-

**Table 3:** Determined parameters of grain distribution for Cli, NaA, and NaX.

Material	Statistical Parameters of Grain Distribution		
	d(0.1) (µm)	d(0.5) (µm)	d(0.9) (µm)
Cli	3.40	26.7	97.8
NaA	3.21	17.1	67.8
NaX	6.56	15.7	33.6

**Table 4:** Parameters estimated for the pseudo-first-order model, pseudo-second-order model, and intra-particle diffusion model for 2,4-D adsorption on all modified materials.

Material	q_e(exp)_ (mg g^−1^)	Pseudo-First-Order Model			Pseudo-Second-Order Model			Intra-Particle Diffusion Model		
		q_e_ (mg g^−1^)	k_1_ (min^−1^)	R^2^	q_e_ (mg g^−1^)	k_2_ (g mg^−1^ min^−1^)	R^2^	k_i_ (mg g^−1^ min^−1/2^)	C (mg g^−1^)	R^2^
Cli_CTAB-0.05_	27	2	0.074	0.8514	14	0.0356	0.9999	0.88	8.08	0.9993
Cli_CTAB-0.1_	6	1	0.043	0.9137	3	0.0752	0.9995	0.23	1.16	0.9970
NaA_CTAB-0.05_	20	2	0.049	0.8404	11	0.0342	0.9999	0.88	5.03	0.9929
NaA_CTAB-0.1_	3	1	0.035	0.9406	2	0.1008	0.9993	0.13	0.87	0.9755
NaX_CTAB-0.05_	26	1	0.036	0.9319	11	0.0353	0.9998	0.59	7.62	0.9763
NaX_CTAB-0.1_	5	1	0.050	0.9138	3	0.0867	0.9999	0.28	1.51	0.9968

**Table 5:** Thermodynamic parameters calculated for 2,4-D adsorption onto zeolites (Cli, NaA, NaX) modified with CTAB.

Material	Temperature (°C)	ΔG° (kJ mol^−1^)	ΔH° (kJ mol^−1^)	ΔS° (J K^−1^ mol^−1^)
Cli_CTAB-0.05_	25	−30.01	−0.0022	100.6
	35	−31.01		
	45	−32.02		
Cli_CTAB-0.1_	25	−23.61	−0.0057	79.2
	35	−24.40		
	45	−25.19		
NaA_CTAB-0.05_	25	−29.21	−0.0015	98.0
	35	−30.19		
	45	−31.17		
NaA_CTAB-0.1_	25	−24.18	−0.0045	81.1
	35	−24.99		
	45	−25.80		
NaX_CTAB-0.05_	25	−27.81	−0.0026	93.3
	35	−28.75		
	45	−29.68		
NaX_CTAB-0.1_	25	−26.10	−0.0063	87.5
	35	−26.97		
	45	−27.85		

**Table 6:** Comparison of the effectiveness of obtained adsorbents with other reported materials for 2,4-D removal.

Material	pH	Mass of Material (mg)	Initial 2,4-D Concentration (mg L^−1^)	Adsorption Capacity (mg g^−1^)	Reference
CTAB-modified fly ash-based zeolite X	5	20	4	~0.4	[45]
CTAB-modified fly ash-based zeolite X	without adjusting (4.7)	20	8	~0.6	[46]
CTAB-modified fly ash-based zeolite A and carbon composite	5	20	4	~0.6	[45]
CTAB-modified fly ash-based zeolite A	5	20	4	~0.65	[45]
CTAB-modified fly ash-based zeolite X and carbon composite	5	20	4	~1.4	[45]
CTAB-modified fly ash-based zeolite X and carbon composite	without adjusting (4.7)	20	8	~1.8	[46]
Fe_3_O_4_ magnetic nanoparticles modified with CTAB	9	~175	200	4.9	[101]
Natural zeolite modified with CTAB	6.7	200	50	8.0	[44]
Fish scales-derived carbon/apatite composite	6.5	20	50	11.1	[102]
Raw sterile bract of *Araucaria angustifolia*	2	500	100	18.95	[103]
Cobalt-grafted activated carbon from date pits and stems	2	100	150	31	[104]
Amino-functionalized poly (glycidyl methacrylate)	3.5	50	100	99.45	[105]
Aminosilane-grafted mesoporous carbon	3	20	150	191	[106]
Quaternary amine anionic-exchange MOF UiO-66	without adjusting	2	80	279	[107]
Cli_CTAB-0.05_	4	50	75	27	This work
NaX_CTAB-0.05_	4	50	75	26	This work

**Table 7:** The non-linear Langmuir, Freundlich, and Temkin model parameters for 2,4-D adsorption onto analyzed samples.

Material	q_e(exp)_ (mg g^−1^)	Langmuir			Freundlich			Temkin		
		q_max_ (mg g^−1^)	K_L_ (L mg^−1^)	R^2^	K_F_ (mg g^−1^ (L mg^−1^)^1/n^)	1/n	R^2^	K_T_ (L g^−1^)	b_T_ (kJ mol^−1^)	R^2^
Cli_CTAB-0.05_	27	28	0.407	0.6178	16.20	0.138	0.8802	71.16	0.730	0.6146
Cli_CTAB-0.1_	6	9	0.048	0.9964	0.86	0.529	0.9613	0.50	1.266	0.9930
NaA_CTAB-0.05_	20	22	0.360	0.9314	10.84	0.172	0.8313	17.86	0.793	0.9172
NaA_CTAB-0.1_	3	4	0.086	0.9990	0.76	0.408	0.9550	0.79	2.541	0.9971
NaX_CTAB-0.05_	26	28	0.216	0.9506	9.95	0.256	0.9372	3.52	0.480	0.9496
NaX_CTAB-0.1_	5	6	0.154	0.9923	1.52	0.316	0.8634	1.49	2.077	0.9787

## Data Availability

The data supporting reported results are provided upon request from the corresponding author.
